# Performance of Zernike polynomials in reconstructing raw-elevation data captured by Pentacam HR, Medmont E300 and Eye Surface Profiler

**DOI:** 10.1016/j.heliyon.2021.e08623

**Published:** 2021-12-18

**Authors:** Yueying Wei, Bernardo T. Lopes, Ashkan Eliasy, Richard Wu, Arwa Fathy, Ahmed Elsheikh, Ahmed Abass

**Affiliations:** a Department of Chemical Engineering and Applied Chemistry, University of Toronto, Toronto, Ontario, Canada; b Department of Mechanical, Materials and Aerospace Engineering, School of Engineering, University of Liverpool, Liverpool, UK; c Department of Civil Engineering and Industrial Design, School of Engineering, University of Liverpool, Liverpool, UK; d Department of Ophthalmology, Federal University of Sao Paulo, Sao Paulo, Brazil; e Brighten Optix Corporation, Shilin District, Taipei City, Taiwan; f Wirral Grammar School for Girls, Bebington, Wirral Peninsula, UK; g School of Biological Science and Biomedical Engineering, Beihang University, Beijing, China; h National Institute for Health Research (NIHR), Biomedical Research Centre at Moorfields, Eye Hospital NHS Foundation Trust and UCL Institute of Ophthalmology, London, UK; i Department of Production Engineering and Mechanical Design, Faculty of Engineering, Port Said University, Egypt

**Keywords:** Corneal tomography, Pentacam, Zernike polynomials, Signal processing, Curve fitting

## Abstract

**Purpose:**

To investigate the capability of Zernike polynomials fitting to reconstruct corneal surfaces as measured by Pentacam HR tomographer, Medmont E300 Placido-disc and Eye Surface Profiler (ESP).

**Methods:**

The study utilised a collection of clinical data of 527 participants. Pentacam HR raw elevation data of 660 eyes (430 healthy and 230 keratoconic) were fitted to Zernike polynomials of order 2 to 20. Same analyses were carried out on 158 eyes scanned by Medmont E300 Placido-disc and 236 eyes were scanned by ESP for comparison purposes. The Zernike polynomial ​fitting was carried out using a random 80% of each individual eye surface's data up to a corneal radius of 5 mm and the root means squared fitting error (RMS) was calculated for the unused 20% portion of the surface data. The process was carried out for the anterior and posterior surfaces of the corneal measurements of the Pentacam HR and the anterior surfaces only with the ESP and the Medmont E300 measurements.

**Results:**

Statistical significances in reduction of RMS were noticed up to order 14 among healthy participants (p < 0.0001 for right eyes, p = 0.0051 for left eyes) and up to order 12 (p < 0.0001 for right eyes, p = 0.0002 for left eyes) in anterior surfaces measured by the Pentacam. Among keratoconic eyes, statical significance was noticed up to order 12 in both eyes (p < 0.0001 for right eyes, p = 0.0003 for left eyes). The Pentacam posterior corneal data, both right and left, healthy and keratotic eyes recorded significance (p < 0.0001) in reduction of RMS up to order 10 with same RMS values of 0.0003 mm with zero standard deviation. RMS of fitting Zernike polynomials to Medmont data up to order 20 showed a consistent reduction in RMS with the increase of the fitting order with no rise at high fitting orders. Minimum RMS = 0.0047 ± 0.0021 mm, 0.0046 ± 0.0019 mm for right and left eyes respectively were recorded at order 20 and were more than 15 times the minimum RMS of the Pentacam. RMS of fitting Zernike polynomials to ESP data also showed a consistent reduction in RMS with the increase of the fitting order with no sign of any rise at high fitting orders. Similar to the Medmont, minimum RMS of 0.0005 ± 0.0003 mm, 0.0006 ± 0.0003 mm was recorded at order 20 for right and left eyes respectively and was 2 times the minimum RMS of the Pentacam for right eyes and 1.7 times the minimum RMS of the Pentacam for left eyes.

**Conclusions:**

Orders 12 and 10 Zernike polynomials almost perfectly matched the raw-elevation data collected from Pentacam for anterior and posterior surfaces, respectively for either healthy or keratoconic corneas. The Zernike fitting could not perfectly match the data collected from Medmont E300 and ESP.

## 1 Introduction

Although several instruments reconstruct anterior eye features in the market with good repetitions in terms of accuracy and repeatability, the common recommendation from the literature is not to use measured values interchangeably among these instruments [1, 2]. Because these instruments use different approaches and different mathematical algorithms to reproduce the corneal topography and tomography, there is no surprise that their final readings are not always comparable [3, 4]. Therefore, understanding the theory and the data handling in each device, hence choosing a suitable mathematical algorithm to reconstruct the measured surfaces would reduce the differences among devices when used to evaluate the same phenomenon. The Pentacam captures sets of cross-sectional images using the Scheimpflug camera, while the Medmont Placido-disc analyses the reflected image of concentric rings, and the Eye Surface Profiler (ESP) captures sinusoidal grating projected images using a charge-coupled device (CCD) camera. Due to these differences, the measured object does not directly represent corneal topography or tomography. Therefore, post-measurement digital signal processing (DSP) procedures are required where the measured data sets are treated in certain ways to represent the anterior eye topography or tomography. Hence refractive power maps and other outputs that eye clinicians use for their diagnosis of eye disorders are influenced by these analyses. Among many other aspects, DSP involves enhancement, representation, reconstruction and, in some cases, interpretation of signals.

Typically, to protect their intellectual property (IP) [5], manufacturers do not always provide full detailed information about the way their instruments process the measured data, therefore, this part of the post-measurement processing is usually unseen by the users and hence, its effect cannot be evaluated directly with conventional approaches [6]. In addition, software-related concerns in medical devices are not rare and could influence health care [7]. Therefore, the current study uses a reverse engineering approach to investigate the post-measurement DSP algorithm in three different instruments and evaluate its effect on the instruments’ measurements. The study investigates the prospect of the use of Zernike polynomial to fit the raw-elevation data and how this possibility could be accounted for or even used by engineers who are using Zernike polynomial to fit Pentacam raw elevation data for the purposes of modelling corneal surfaces or to carry out wavefront analyses.

## 2 Materials and methods

### 2.1 Participants

In this record review analysis, no participant had been recruited specially for this study, therefore fully anonymised secondary data was used. The study utilised a collection of clinical data that has been used in various previous studies [8, 9, 10, 11, 12,13, 14, 15, 16, 17, 18] where only valid data, in terms of quality, were selected to be processed. Recorded data for individuals who were suffering from ocular diseases or have a history of trauma or ocular surgery, including Asian upper blepharoplasty, were excluded. Additionally, those with intraocular pressure (IOP) higher than 21 mmHg as measured by the Goldmann Applanation Tonometer, soft contact lens wear until less than two weeks before measurement, or rigid gas-permeable (RGP) contact lens wear until less than four weeks before measurements were excluded.

In order to avoid bias, right and left eyes were always treated independently from each other, and no merging data technique was applied in this work. According to the University of Liverpool's Policy on Research Ethics, ethical approval was unnecessary for secondary analysis of fully anonymised data. Nevertheless, the study followed the tenets of the Helsinki Declaration.

### 2.2 Pentacam HR data

The study used recorded data of both eyes of 330 healthy participants aged 35.6 ± 15.8 years and 230 Keratoconic participants aged 31.6 ± 10.8 years. Participants were selected from referrals to Hospital de Olhos Santa Luzia, Maceio, Alagoas, Brazil. Clinical tomography data has been collected from both eyes of participants using the Pentacam HR (OCULUS Optikgeräte GmbH, Wetzlar, Germany). Pentacam HR raw elevation data for the anterior surface were exported in comma-separated values (CSV) format and analysed using custom-built MATLAB codes (MathWorks, Natick, USA). Data was extracted over a mesh grid covering -7 to 7 mm in 141 steps in both nasal-temporal and superior-inferior directions with missing elevation values around corners and edges set to NaN which stands for “Not a Number”. The effect of missing elevation values was automatically avoided arithmetically and logically during the analyses. This is because any arithmetic operation in MATLAB that involves a NaN produces a NaN as well. Furthermore, MATLAB logical operations (true-false) involving NaNs always return as false.

### 2.3 Medmont E300 data

Medmont E300 Placido-disc elevation data for the corneal anterior surface were exported in Microsoft Excel spreadsheet (XLSX) format and analysed using custom-built MATLAB codes. Data was extracted over a mesh grid covering -6 to 6 mm in 50 steps in both nasal-temporal and superior-inferior directions with missing elevation values around the edges set to a big negative value of -5×10^20^.

Both right and left eye anonymised topography data were extracted from the recorded data of 79 Caucasians (158 eyes); 41 females and 38 males aged 43.3 ± 11.5. The eye surface scan process was carried out using the Medmont E300 corneal topographer (Medmont International, Nunawading, Australia).

### 2.4 ESP data

Both right and left eye anonymised topography data were extracted from the recorded data of both eyes of 125 Taiwanese Asian and 118 Caucasian subjects aged 22–67 years (38.5 ± 7.6). Groups were properly gender-balanced (Asians: 66 females and 59 males; Caucasians: 63 females and 55 males). The eye surface scan process was carried out using ESP, a non-contact corneo-scleral topographer, Eaglet Eye BV, AP Houten, The Netherlands).

The data was exported from the ESP software in MATLAB binary data container format (∗.mat) where the characteristics of eyes, as measured by the ESP system, were extracted and processed. The eye surface data was processed by custom-built MATLAB codes independent from the built-in ESP software. Data was extracted over a mesh grid covering -10 to 10 mm in 700 steps in the nasal-temporal direction, and -8 to 8 mm in 800 steps in the superior-inferior direction with missing elevation values around the edges set to NaN.

### 2.5 Corneal surfaces fitting

**Figure 1:**
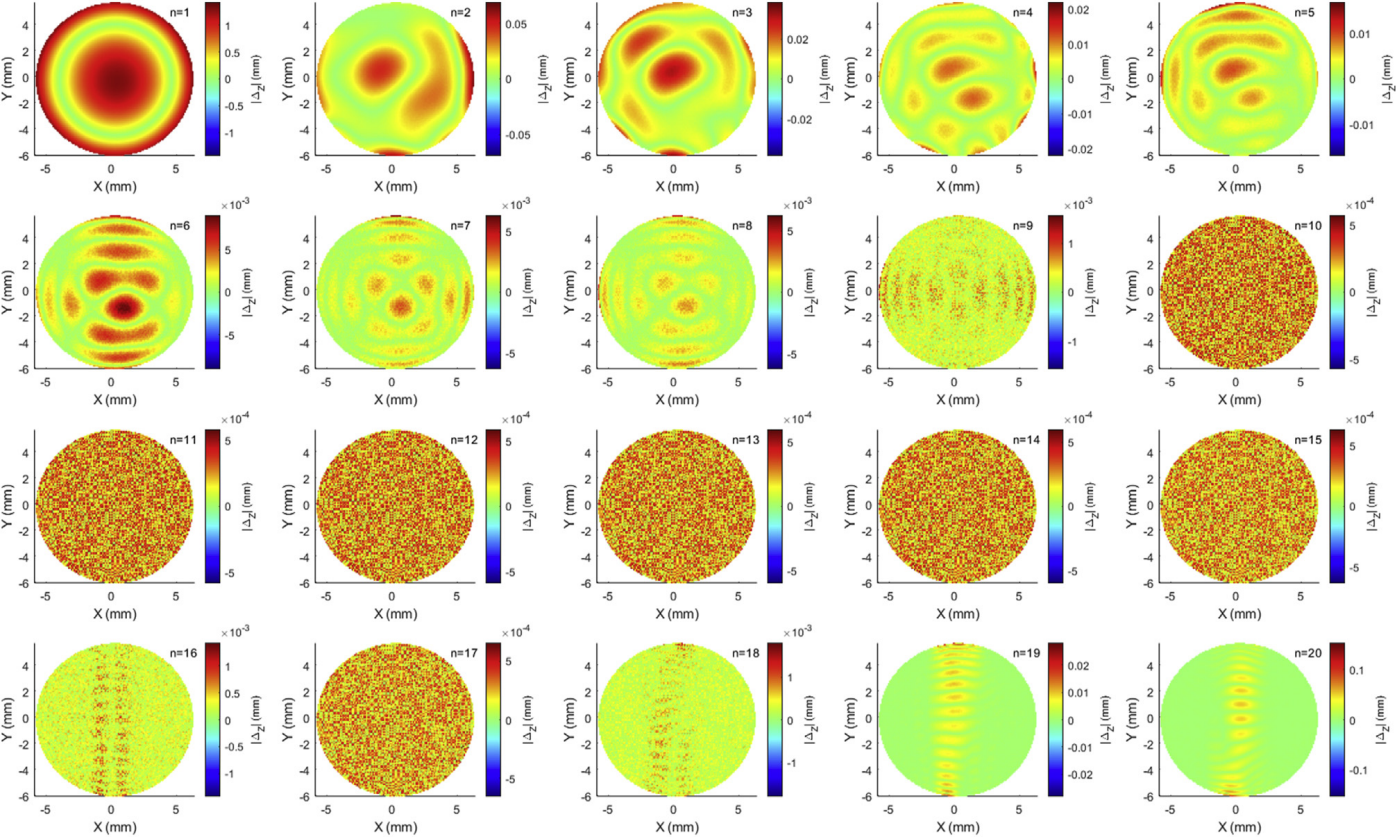
Zernike polynomial absolute fitting error Δ_z_ = |Z_fit_-Z_surf_| for the anterior corneal surface of 27 years old keratotic female participant measured by the Pentacam HR tomographer.

Three-dimensional curve fitting is a process that aims to reconstruct a surface through a parametric mathematical expression or nonparametric method that best suits a cloud of data points. In the current study, Zernike polynomials were used as parametric mathematical expressions that are capable of reconstructing corneal surfaces. As each one of the three instruments used in this study is able to cover the cornea to different diameters, a maximum radius of 5 mm was used in the fitting exercise for all instruments, Figure 1. Any surface data beyond this maximum radius were set to NaN, hence disregarded in these analyses. Therefore, the surface grid is centred around the corneal apex, then the radius of each point in $R_{g}$ the grid is calculated in Eq. (1) as

(Eq.1) $$r=\sqrt{X_{g}^{2}+Y_{g}^{2}}\;\;\&\;\;Z_{g}\left(r>5\right)=NaN$$

where *X*_*g*_ and *Y*_*g*_ represent the grid points in the nasal-temporal and superior-inferior directions, respectively and *Z*_*g*_ is the corneal raw elevation.

Once the data within the 5 mm radius was identified, the Zernike polynomial fit sequence was carried out with orders 1 to 20 using the minimum least squared error method and the root-mean-square (RMS) error values were recorded for each fit. At this point, a normalised form of the radius r was calculated in Eq. (2) as

(Eq. 2) $$r_{n}=\frac{r}{r_{max}},\text{where ​}r_{max}=5mm$$

Zernike polynomials used the polar coordinates $\left(r_{n},\theta \right)$ and the relevant raw elevation data obtained for each cornea to express the radial distance ρ as presented in Eq. (3).

(Eq. 3) $$\rho =\sum_{n=0}^{order}\sum_{m=-n:2:n}Z_{n}^{m}\left(r,\theta \right)C_{n}^{m}\left(\theta \right)$$

where Zernike term is represented by Eq. (4) as

(Eq. 4) $$Z_{n}^{m}\left(r,\theta \right)=\left\{ \begin{array}{ll} R_{n}^{\left|m\right|}\cos\left(m\theta \right) & m>0\\ R_{n}^{\left|m\right|}\sin\left(m\theta \right) & m<0\\ R_{n}^{\text{0}} & m=0 \end{array}\right.$$

with the radial polynomial $R_{n}^{\left|m\right|}$ defined in Eq. (5) as

(Eq. 5) $$R_{n}^{\left|m\right|}=\sum_{i=0}^{\frac{n-\left|m\right|}{2}}\frac{{\left(-1\right)}^{i}\left(n-1\right)!r^{n-2i}}{i!\left(\left(n+\left|m\right|\right)/2-i\right)!\left(\left(n-\left|m\right|/2\right)\right)\text{!}},\left(0\leq r\leq 1\right)$$

Where (r,θ) are the polar coordinates of *X*_*g*_ and *Y*_*g*_, *n* is the radial order of the polynomial, and m is an azimuthal integer index that varies from -n to n for even (m-n) and equals 0 for odd (n-m). The fitting RMS error was calculated twice for every fit during the fitting process, firstly by using the whole surface for fitting and validation, then secondly by randomly selecting 80% of the data points for fitting and the other 20% to calculate the fitting RMS error by Eq. (6) as

(Eq. 6) $$RMS=\sqrt{\frac{\sum_{i=1}^{k}{\left(Z_{i\;fit}-Z_{i\;surf}\right)}^{2}}{k}}$$

where *Z*_*fit*_ is the Zernike fitted surface height and *Z*_*surf*_ is the measured raw elevation surface height and *k* is the number of non-missing data points. In this study, the RMS error represents the squared root of the averaged squared variations between fitted surface height points *Z*_*fit*_ and clinically observed surface height points *Z*_*surf*_. The process was carried out for the anterior and posterior surfaces of the corneal measurements of the Pentacam and the anterior surfaces only with the ESP and the Medmont E300 Placido-disc measurements as both of them measure the corneal anterior surface only.

### 2.6 Statistical analysis

Statistical analysis was performed using MATLAB Statistics and Machine Learning Toolbox (MathWorks, Natick, USA). The null hypothesis probability (p) at 95% confidence level was calculated to compare each set of RMS errors when a corneal surface was fitted to Zernike polynomial with a certain order with the set of RMS errors when the same corneal surface was fitted to Zernike polynomial with one order less. Initially, the one-sample Kolmogorov-Smirnov test was used to make sure that each set of RMS errors follows a normal distribution, then the two-sample t-test was used to investigate the significance between pairs of data sets to check whether the results represent independent records. The probability p is an element of the period [0,1] where values of p higher than 0.05 indicate the validity of the null hypothesis and values less than or equal to 0.05 indicate the invalidity of the null hypothesis, hence statistical significance [19].

## 3 Results

**Figure 2:**
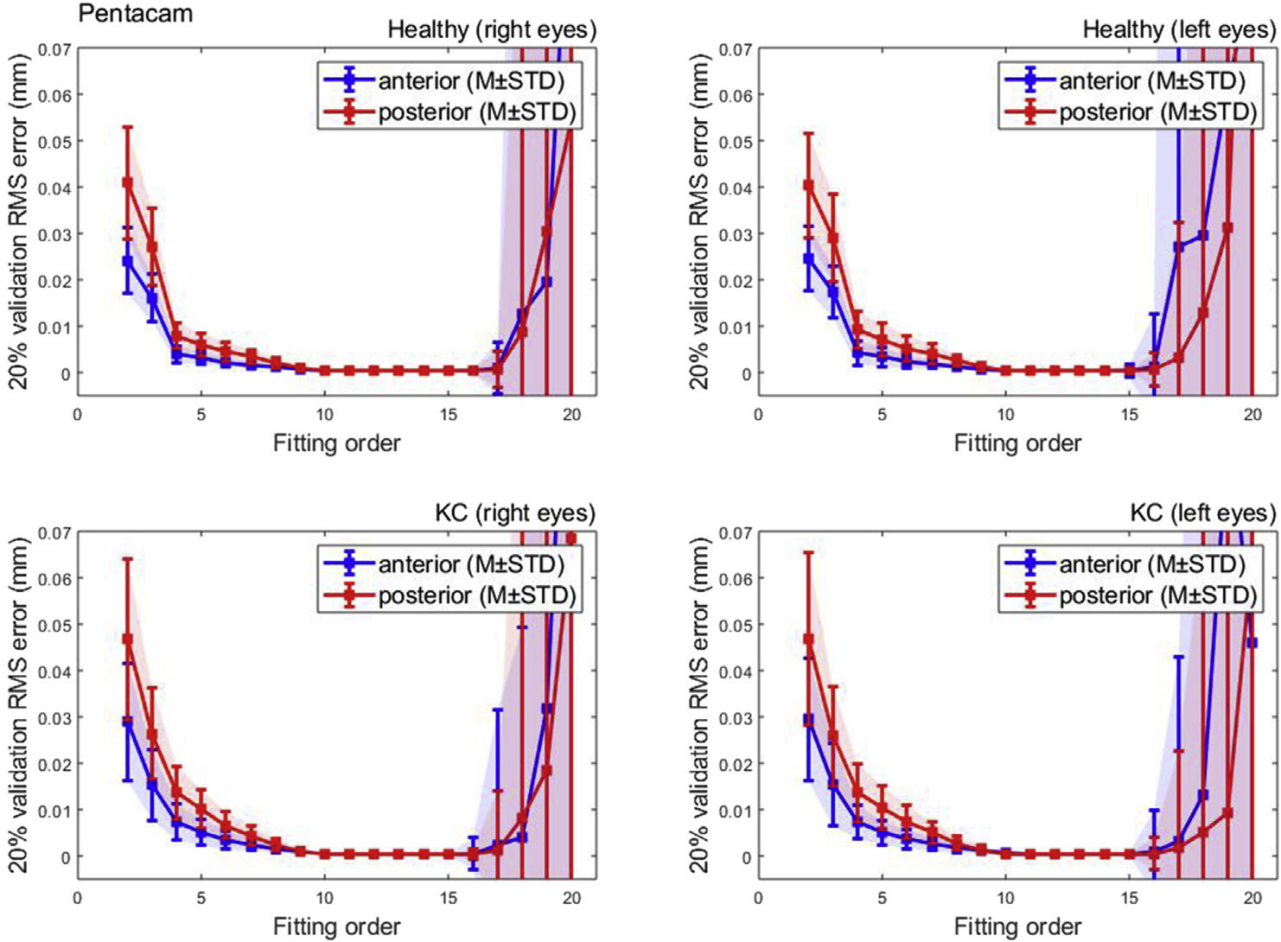
Pentacam HR Zernike polynomial fitting RMS error with 20% validation for healthy and keratotic populations.

The results showed that the Pentacam anterior surface Zernike polynomial fitting RMS decreased with the increase of the fitting order, Table 1, however, the small values of the RMS error from order 10 (RMS = 0.0004 ± 0.0001 mm for right eyes, RMS = 0.0005 ± 0.0002 mm for left eyes) to 15 (RMS = 0.0003 ± 0.0001 mm for right eyes, RMS = 0.0004 ± 0.0015 mm for left eyes) were notable in healthy subjects. The same phenomenon was noticed in keratoconic patients between order 10 (RMS = 0.0005 ± 0.0002 mm for right eyes, RMS = 0.0005 ± 0.0002 mm for left eyes) and order 15 (RMS = 0.0003 ± 0.0002 mm for right eyes, RMS = 0.0004 ± 0.0003 mm for left eyes). From fitting order 16, RMS values started to rise exponentially to record 0.1221 ± 0.8218 mm, 0.0837 ± 0.7085 mm, 0.1419 ± 1.6770 mm, 0.2564 ± 0.4612 mm for healthy right and left eyes and keratoconic right and left eyes, respectively, Figure 2.

**Table 1:**
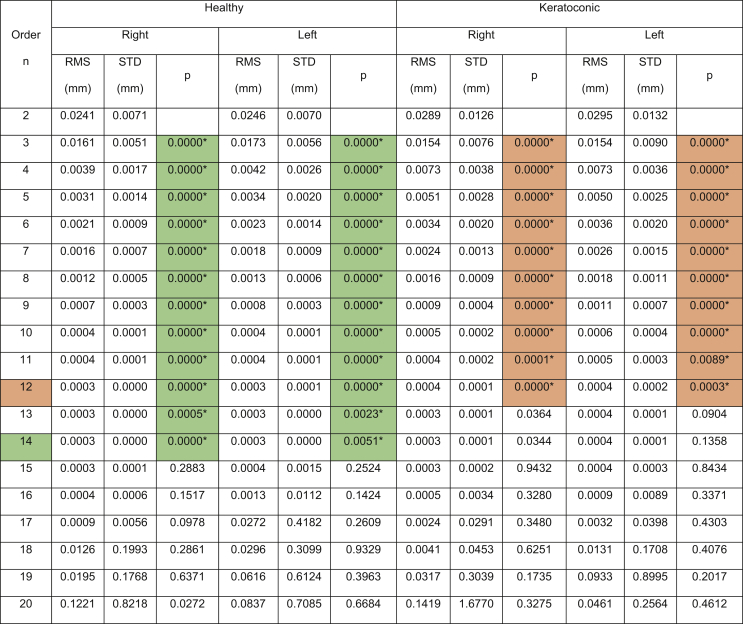
Zernike polynomial fitting RMS for both Pentacam healthy and keratoconic participants’ corneal anterior surfaces.

(∗) Indicates statistical significance.

**Figure 3:**
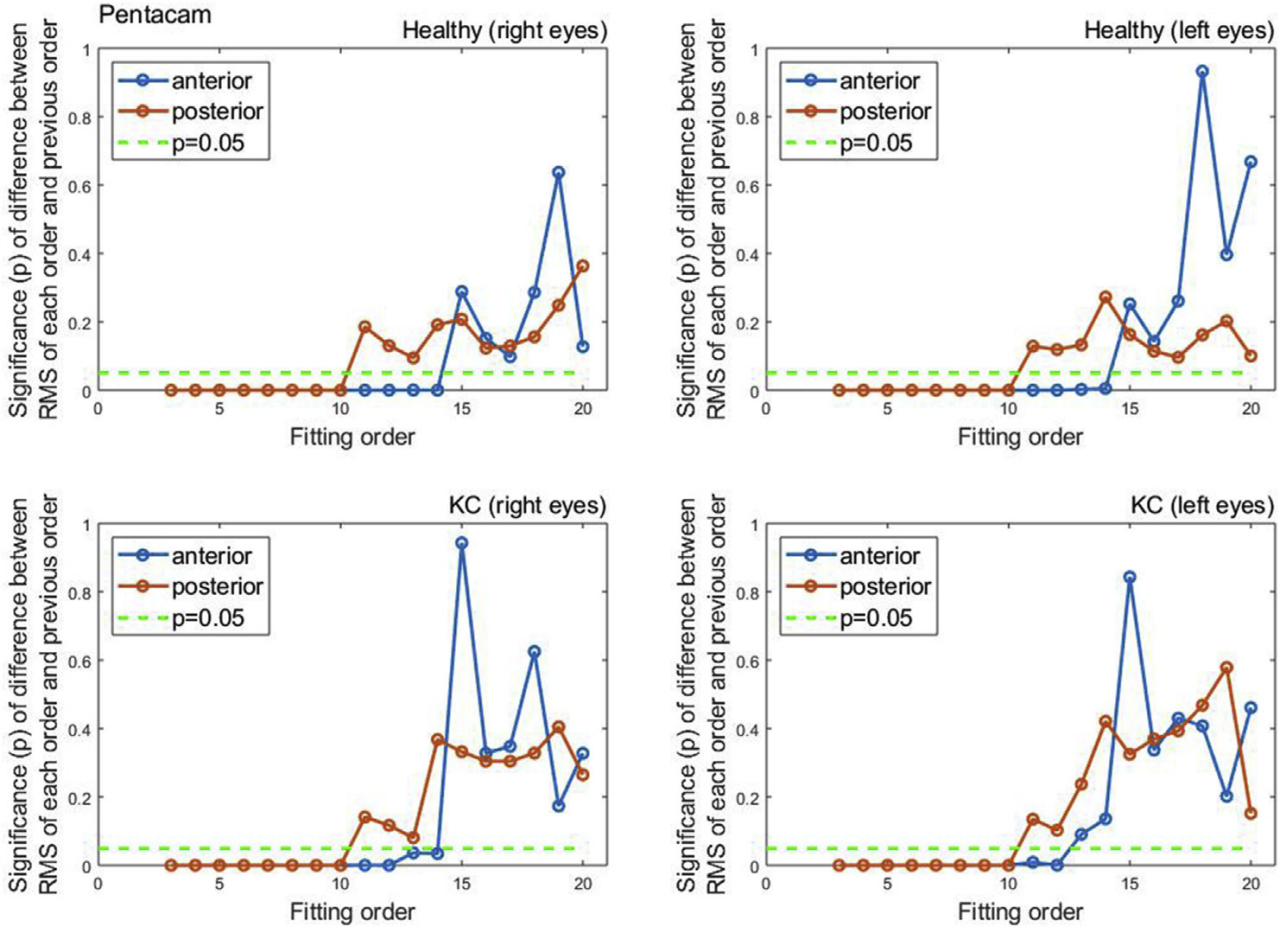
Significance (p) of difference between RMS of each order and previous order among normal and keratoconic cases.

To evaluate the quality of fitting of each order against the previous order, the two samples t-test was used to compare the RMS of each order with the previous order, Figure 3. When the difference in RMS values at each order n compared to the previous order n-1, statistical significances were noticed up to order 14 among healthy participants (p < 0.0001 for right eyes, p = 0.0051 for left eyes) and up to order 12 (p < 0.0001 for right eyes, p = 0.0002 for left eyes). Among keratoconic eyes, statical significance was noticed up to order 12 in both eyes (p < 0.0001 for right eyes, p = 0.0003 for left eyes).

Remarkably, when the corneal posterior surface was investigated in the Pentacam data, both eyes right and left eyes of healthy and keratotic participants recorded significance (p < 0.0001) in fitting RMS up to order 10 with the same RMS values of 0.0003 mm and zero standard deviation for all right, left, healthy and keratotic eyes, Table 2.

**Table 2:**
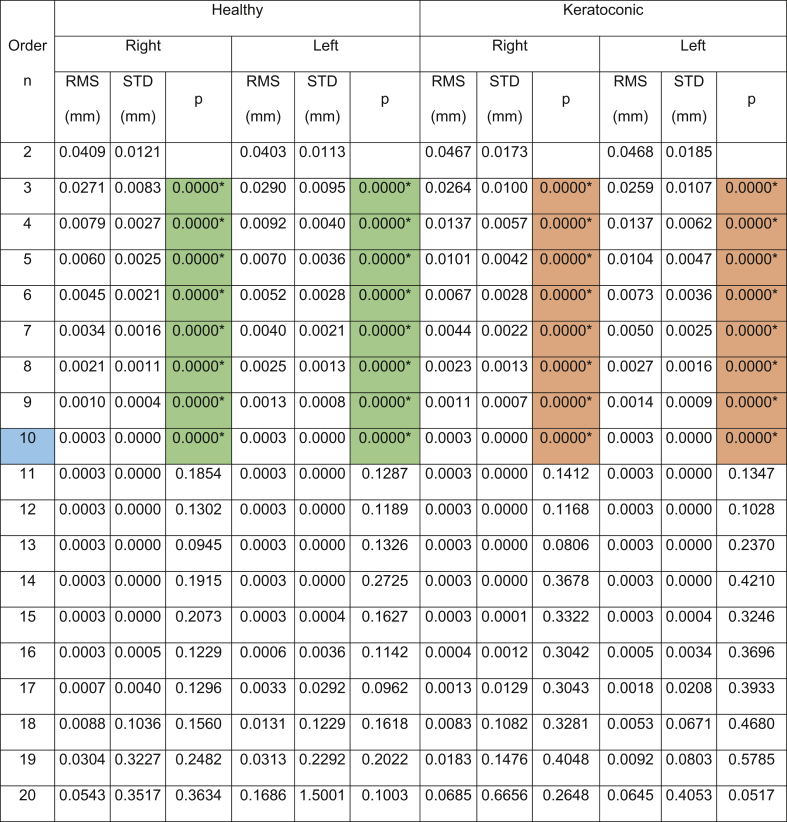
Zernike polynomial fitting RMS for both Pentacam healthy and keratoconic participants’ corneal posterior surfaces.

(∗) Indicates statistical significance.

**Figure 4:**
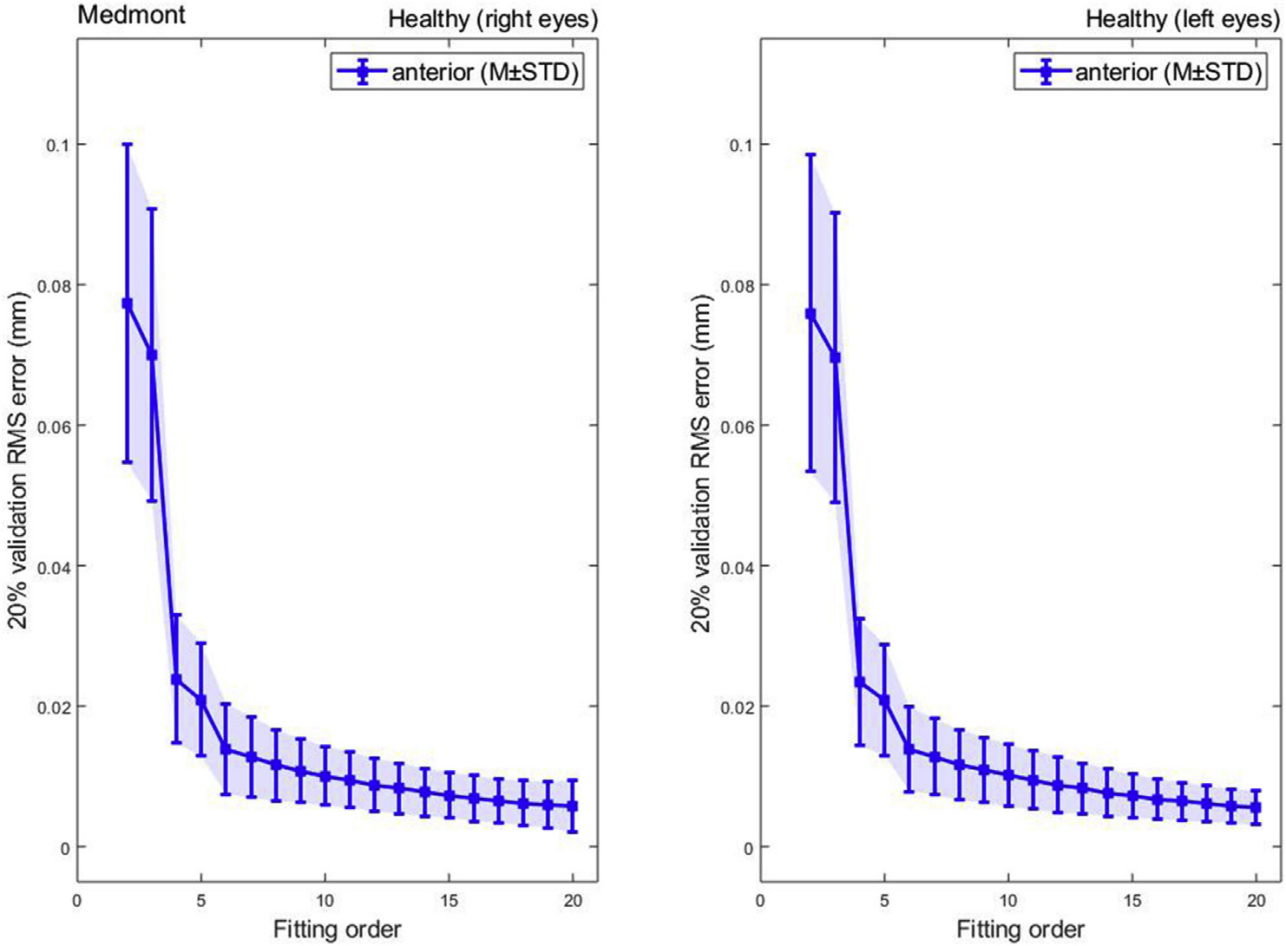
Medmont Zernike polynomial fitting RMS error with 20% validation for a healthy population.

Unlike the Pentacam tomography fitting outcome, RMS of fitting Zernike polynomials to Medmont data up to order 20 showed a consistent reduction in RMS with the increase of the fitting order with no rise at high fitting orders, Figure 4. Minimum RMS = 0.0047 ± 0.0021 mm, 0.0046 ± 0.0019 mm for right and left eyes respectively were recorded at order 20 and were more than 15 times the minimum RMS of the Pentacam, Table 3.

**Table 3:**
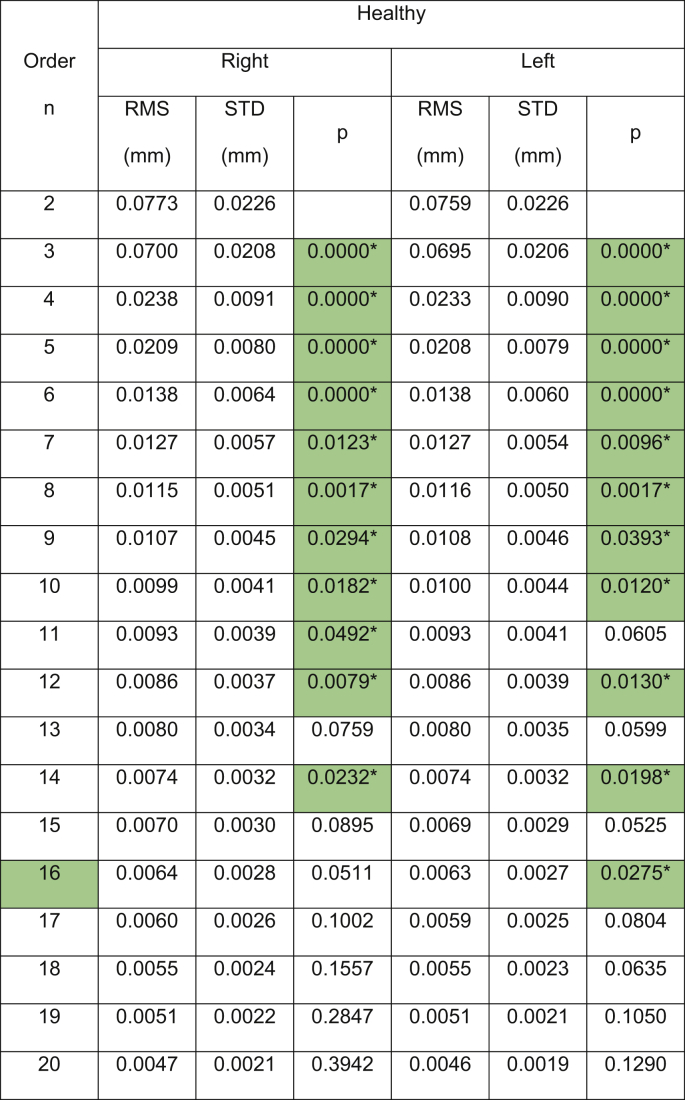
Zernike polynomial fitting RMS for Medmont Placido disc healthy participants.

(∗) Indicates statistical significance.

**Figure 5:**
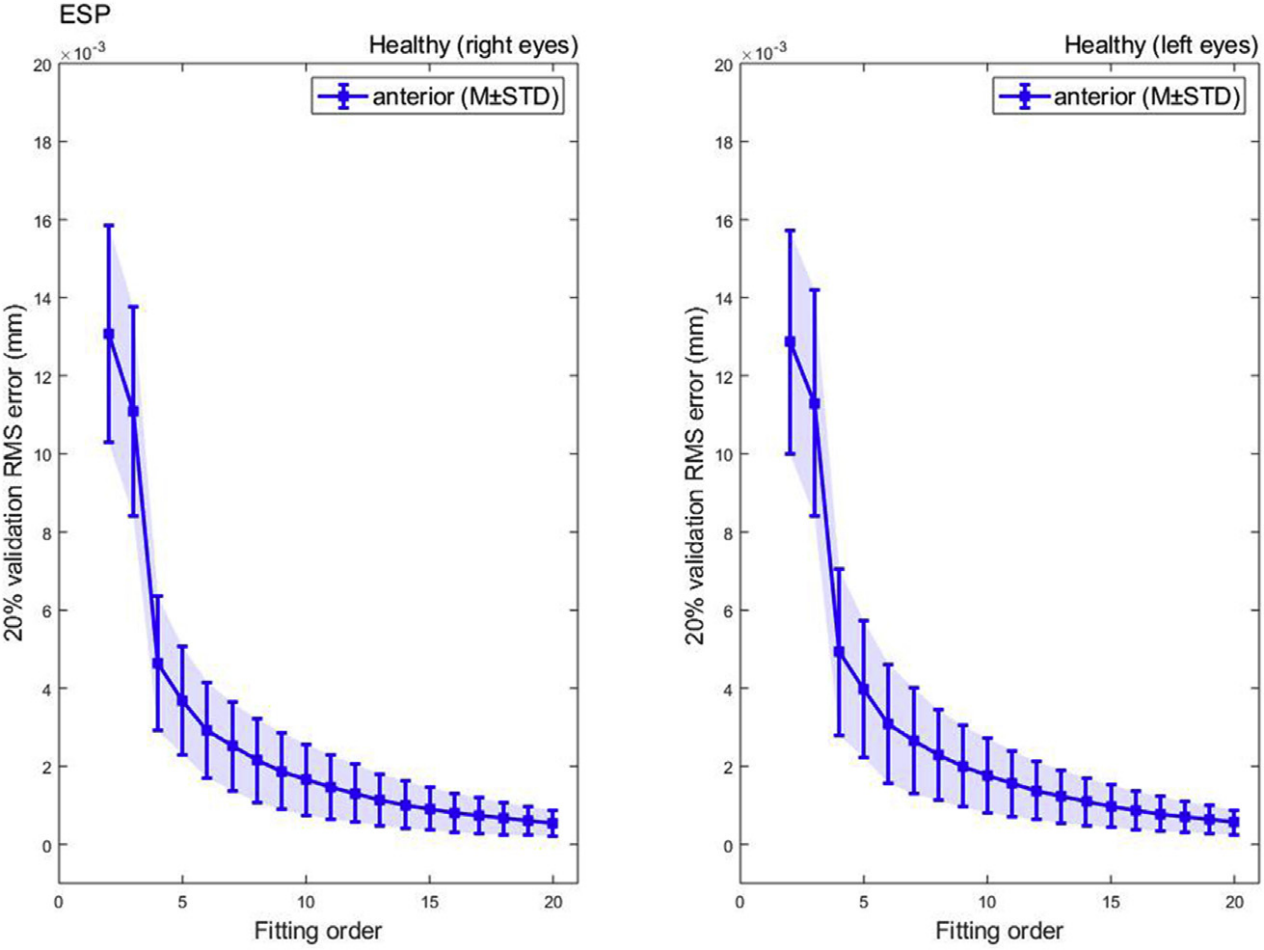
ESP Zernike polynomial fitting RMS error with 20% validation for a healthy population.

Like the Medmont Placido disc, and unlike the Pentacam tomography fitting outcome, RMS of fitting Zernike polynomials to ESP data up to order 20 also showed a consistent reduction in RMS with the increase of the fitting order with no sign of any rise at high fitting orders, Figure 5. Similar to the Medmont, minimum RMS of 0.0005 ± 0.0003 mm, 0.0006 ± 0.0003 mm was recorded at 20 for right and left eyes respectively and was 2 times the minimum RMS of the Pentacam for right eyes and 1.7 times the minimum RMS of the Pentacam for left eyes, Table 4.

**Table 4:**
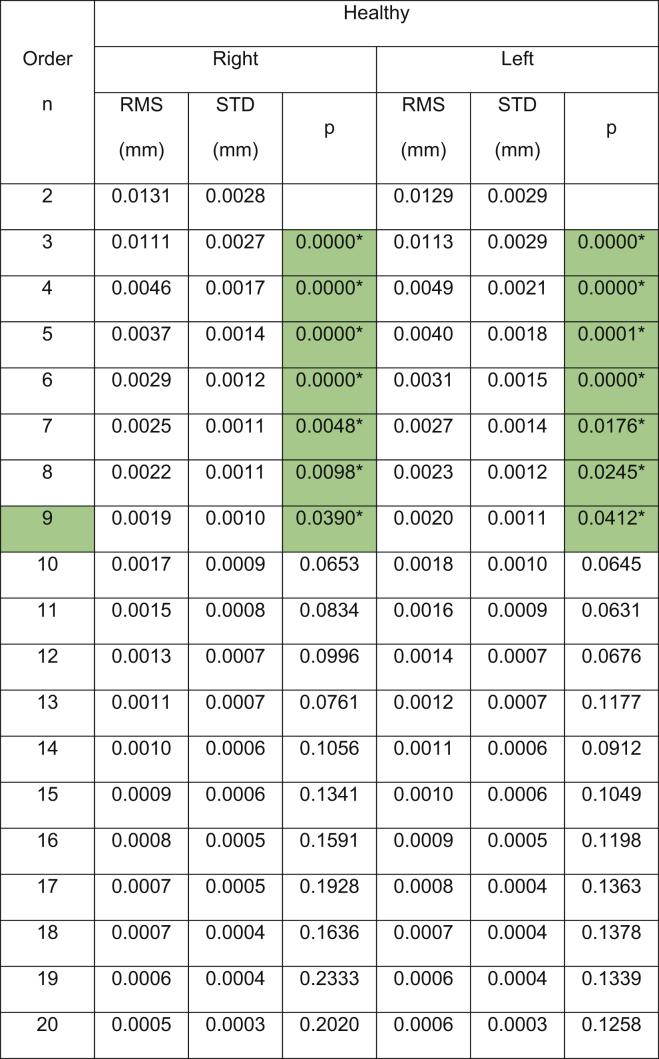
Zernike polynomial fitting RMS for ESP healthy participants.

(∗) Indicates statistical significance.

## 4 Discussion

Although tomographer, topographers and surface profilers are widely accepted in scientific research, some of them do not offer a direct measure of topography. Numerous studies published data collected from Pentacam and several compared its performance to that of other topographers and reported high correlation [3, 20, 21, 22, 23]. It is also important to acknowledge the studies suggested that repeatability of Scheimpflug devices can be lower for the posterior corneal surface than for the anterior corneal surface [24, 25] however, measurements taken with the Pentacam are reported to be repeatable and reproducible when they are obtained with the high-resolution settings and analysed with caution [26].

Placido-disk topography systems have their limitations too. Placido-disc based systems, unlike Pentacam HR, cannot provide measurements for the posterior surface of the cornea. Posterior elevation data were reported to have a significant effect on overall corneal astigmatism magnitude, astigmatism axis [25, 27], optical axis [18] and keratoconus cone location [28]. In addition, they cannot measure the corneal central zone within the first mire ring, and as a result, this region has to be interpolated using a relatively narrow (≅ 60%) corneal surface coverage [29, 30]. They use images obtained from light reflected off the tear film, thus the inconsistent quality of corneal tear film becomes an essential limitation. Moreover, Placido-disk systems data are less accurate when mapping irregular surfaces due to their methodology hypothesis of significant smoothness in the radial direction [31].

Like the other two devices, the ESP has some limitations. It is not possible to use eye profile data without considering a method of removing the edge-effect. The artefacts around the edges are not naturally present features but appear on the measured surface as a result of the instrument limitation, the measurement protocol and the technological limits [12].

The difference between a corneal measured feature and its true value is a measurement error that could be either random, systematic [32] or a combination of both along with other factors. Random errors naturally occur during any measurement because of disturbances such as environmental conditions or electronic noise. The positive element is that random errors have a Gaussian normal distribution, therefore, statistical methods can be effectively used to analyse the measured data and determine the significance of any change in the measured feature regardless of the associated random errors. Systematic errors usually occur as a result of using a miscalibrated instrument or because of the incorrect use by the operator [33]. Although these errors are important to consider, they are not the only artefacts in the corneal structure measurement process. There is something else embedded within the instruments' software packages called DSP. Among many other aspects, DSP involves detection, estimation, coding, transmission, enhancement, analysis, representation, recording, reconstruction, transformation and interpretation of digital signals [34]. With no access to the tomographers and topographers’ built-in pieces of DSP within their software, reverse engineering is one of the best methods for researchers to investigate unseen DSP components. DSP within the output researchers get may affect their interpretation or their understanding of the numerical values produced by eye reconstruction software-driven instruments.

The technique used in this study can be considered a reverse engineering fitting method. The results showed that the posterior corneal surface measured by Pentacam fits perfectly to order 10 Zernike polynomials with a very small RMS (3 × 10^−4^) and zero standard deviation. This finding indicates the possibility of the Pentacam posterior corneal surface being fitted to order 10 Zernike polynomials during the DSP stage. This conclusion is supported by the fact that fitting the posterior surface to orders up to 15 did not record significant reductions in RMS compared to order 10. It is also supported by the fact that both heathy and keratoconic participants data showed the exact trend with no noticeable difference. This indicates that this fitting is potentially a built-in DSP sequence within the Pentacam software.

On the other hand, the anterior surface of the Pentacam fitted very well to order 12 Zernike polynomials in both healthy and keratoconic participants. While healthy eyes still fit well up to order 14, the significance test showed that keratoconic eyes are not recoding improvement in RMS values after order 12. With a standard deviation of nearly zero, there is a strong possibility that a fit of order 12 Zernike polynomials was applied to anterior eye surfaces within the Pentacam DSP stage. The closest study to the current one was presented by Smolek, in 2005, on TMS-1 (Tomey, Inc, AZ, US) corneal topography maps where he concluded that 4^th^ order Zernike polynomial reconstruction was reliable for modelling the normal cornea only, but significantly higher orders are needed for reconstructing abnormal corneal surfaces [35]. However, the current study findings do not endorse 4^th^ order Zernike polynomial reconstruction for Pentacam HR tomographer, Medmont E300 Placido-disc and ESP data. The reverse engineering technique used here showed unique compatibility between the Pentacam elevation data and Zernike polynomials. In addition, the RMS started to rise again after certain order as an indication of an overfitting issue which is known to be associated with polynomial fitting. None of the other two machines showed any rise in RMS as a result of increasing the fitting order.

A possible limitation in this study is not splitting the data according to age groups or ethnic background and not grouping keratoconic groups according to the severity of the disease. As the focus of this study is the DSP within the pieces of the instrument's software, the participants' data were analysed according to the instrument not according to the age groups or the ethnic background. The only exception was analysing the keratoconic Pentacam data separately from the healthy ones to investigate the response of distorted eyes to the Zernike polynomial fitting process. Limitations of not testing keratoconic eyes or even animal eyes will be addressed soon in a future study. Additionally, Zernike polynomials are not the only type of polynomials that could be used to fit corneal surfaces. Tchebichef [36], Krawtchouk [37], Charlier [38], and Meixner polynomials [39] could be used too, however, Zernike polynomials are broadly deemed to be the mathematical base of ocular aberrations [40].

The results suggest using order 10 Zernike polynomial to fit Pentacam posterior corneal surface and order 12 Zernike polynomial to fit Pentacam anterior surface is an ideal option to analysts who are interested in wavefront analyses, high order aberrations, light raytracing, and other applications that require parametric continuous surfaces to operate. Fitting Medmont E300 Placido-disc and ESP to Zernike polynomials is not recommended because of the relatively high RMS associated with this fit, however, if necessary Medmont E300 Placido-disc's topography and ESP's corneal profile could be fitted to Zernike polynomial order 16 and 9 respectively with a consciousness of the possible effect of the fitting error.

## Declarations

### Author contribution statement

Yueying Wei: Performed the experiments.

Bernardo T Lopes & Ashkan Eliasy: Analyzed and interpreted the data.

Richard Wu & Ahmed Elsheikh: Contributed reagents, materials, analysis tools or data.

Arwa Fathy: Performed the experiments; Wrote the paper.

Ahmed Abass: Conceived and designed the experiments; Analyzed and interpreted the data; Contributed reagents, materials, analysis tools or data; Wrote the paper.

### Funding statement

This research did not receive any specific grant from funding agencies in the public, commercial, or not-for-profit sectors.

### Data availability statement

Data included in article/supp. material/referenced in article.

### Declaration of interests statement

The authors declare no conflict of interest.

### Additional information

No additional information is available for this paper.
